# Developing a socio-ecological framework for promoting physical activity among Chinese children and adolescents: a Delphi–AHP study

**DOI:** 10.3389/fpubh.2025.1700544

**Published:** 2025-12-02

**Authors:** Lin Kong, Xiqian Zhang, Xinyu Chen, Junkai Zhang, Yanguo Yang, Mingming Guo

**Affiliations:** 1 School of Physical Education, Guangdong University of Education, Guangzhou, Guangdong, China; 2 Department of Physical Education, College of Education for the Future, Beijing Normal University, Zhuhai, Guangdong, China; 3 College of Physical Education, Shanghai Normal University, Shanghai, China

**Keywords:** physical activity, children, adolescents, Delphi method, analytic hierarchy process, socio-ecological framework, China

## Abstract

**Purpose:**

Insufficient physical activity (PA) among children and adolescents poses a serious public health challenge in China, with over 84% failing to meet the World Health Organization’s daily recommendations. Despite national policies promoting PA, a lack of localized, evidence-based frameworks has hindered effective implementation.

**Method:**

Guided by socio-ecological theory, a modified Delphi method was conducted with 17 experts across two rounds to refine framework dimensions and indicators. The Analytic Hierarchy Process (AHP) was then applied to calculate indicator weights.

**Results:**

The final framework comprised four dimensions—school leadership, family involvement, community support, and societal collaboration—encompassing 17 primary indicators and 58 secondary indicators. Weight analysis highlighted schools as the most influential domain (54.37%), followed by families (23.14%), communities (12.11%), and societal collaboration (10.38%).

**Conclusion:**

This study is the first to establish a localized framework for PA promotion among Chinese children and adolescents, offering clear stakeholder responsibilities and weighted indicators to guide evidence-based interventions. While grounded in China’s unique sociocultural setting, the methodology may inform similar efforts internationally. Future research should focus on empirical validation and development of standardized assessment tools based on this framework.

## 1 Introduction

The World Health Organization (WHO) has identified insufficient physical activity (PA) as a major public health concern, attributing approximately 6% of all global deaths each year to this risk factor. Insufficient PA is consistently ranked among the top ten leading contributors to global mortality (1). Alarmingly, physical inactivity has reached epidemic proportions, with an estimated 31.1% of the world’s population classified as inactive (2). Such widespread inactivity not only exacerbates the risk of developing non-communicable diseases but also generates a substantial economic and healthcare burden at both individual and societal levels (3, 4).

PA during childhood and adolescence has long-term implications for activity levels in adulthood. Establishing healthy movement behaviors early in life may extend into adulthood, conferring continuous health benefits across the life course (5). Therefore, cultivating PA habits during childhood and adolescence is of critical importance. The WHO recommends that children and adolescents aged 5–17 years engage in at least 60 min of moderate-to-vigorous physical activity (MVPA) daily (1). However, global data from 2022 indicated that 81% of children and adolescents engaged in less than 60 min of MVPA per day (2). In comparison, the situation in China is even more concerning, with 84% of children and adolescents failing to meet the recommended daily 60 min of MVPA (2).

In 2018, the WHO released the Global Action Plan on Physical Activity 2018–2030, providing guidance for member states to accelerate and expand efforts to increase population-level PA (6). Following this initiative, many countries and regions—including the United States, the United Kingdom, Canada, Japan, Australia, Finland, New Zealand, and Singapore—have introduced their own PA guidelines and actionable intervention programs (7–10). China also issued the Guidelines on Physical Activity for Children and Adolescents in 2018 (11). However, owing to the limited availability of practice-based research evidence specific to Chinese children and adolescents, the development of the Chinese guidelines has relied largely on synthesizing research findings from other countries (12).

This gap further underscores the importance of conducting localized, evidence-based research on PA among Chinese children and adolescents. A systematic framework and feasible, practice-oriented strategies are essential foundations for such research (12). To address this urgent need, several critical questions must first be clarified: Within the Chinese sociocultural context, what roles do schools, families, communities, and governments play in promoting PA among children and adolescents? What specific actions can each stakeholder take? What are the key priorities and strategies? How can a systematic framework for PA promotion be used to design targeted interventions for children and adolescents? Furthermore, how can the policy translation pathway of “framework development – model construction – action implementation” enhance the effectiveness of PA interventions? Therefore, constructing a scientific, systematic, and actionable framework for promoting PA among children and adolescents is not only a pressing necessity for advancing PA promotion in China but also a crucial measure for aligning with global health strategies and responding to the demands of the current era.

Against this backdrop, the present study aims to develop a comprehensive framework for promoting PA among children and adolescents that is tailored to China’s sociocultural context and aligned with developmental characteristics and practical needs, using an modified Delphi method. Specifically, the study systematically reviewed domestic and international policy documents and practical initiatives related to the promotion of PA in children and adolescents. Grounded in the current status of PA promotion in China and guided by socio-ecological theory, the study employed the Delphi method and analytic hierarchy process (AHP) to construct a multi-level framework encompassing schools, families, communities, and society. This framework is intended to provide a quantifiable, traceable, and optimizable action guide for promoting PA among Chinese children and adolescents.

## 2 Method

### 2.1 Study design

This study followed a series of systematic steps to develop a framework for promoting PA among Chinese children and adolescents. First, a preliminary framework was established based on a review of relevant literature and expert telephone interviews. These sources helped identify key developmental characteristics, environmental contexts (e.g., school and community settings), and health-related needs (e.g., physical fitness and motor development) pertinent to PA promotion in this population.

Subsequently, an modified Delphi method was employed to refine the framework through two iterative rounds, screening and optimizing its components, primary indicators, and secondary indicators. The experts consulted during the telephone interview stage were the same as those who later participated in the Delphi survey. Finally, the AHP was applied to calculate the weight of each indicator.

### 2.2 Literature search

The literature review aimed to provide the theoretical foundation and an initial pool of indicators for constructing the framework. This was achieved by synthesizing studies published in English or Chinese that focused on the concepts, characteristics, influencing factors, intervention strategies, or frameworks of PA.

The literature was retrieved from four electronic databases: CNKI, EBSCO, Web of Science Core Collection, and ProQuest. The search period for English-language studies covered January 2001 to December 2024, while the search period for Chinese-language studies spanned January 2012 to December 2024. The keywords used included “PA promotion,” “physical health promotion,” “health promotion,” “PA intervention,” and “PA for children and adolescence”.

### 2.3 Expert selection

Following the expert selection strategies employed by Zhang et al. and Tao et al., experts were identified through systematic searches of leading domestic and international academic journals, official websites of relevant professional organizations, and peer recommendations (13, 14).

Eligibility criteria required that experts: (1) held at least an associate professor position (or equivalent professional title); (2) had a minimum of 5 years of experience in physical education, health promotion, or related fields; (3) had demonstrable research or practical expertise in PA promotion; and (4) were able to complete all Delphi rounds independently. Exclusion criteria included direct collaboration with the authors in the past three years or failure to complete all Delphi rounds.

A purposive sampling strategy was employed to ensure diversity across academic disciplines and geographic regions. Experts were recruited from universities, research institutes, governmental organizations, and schools. All invited experts were assured of anonymity, and individual responses were treated confidentially.

Ultimately, 17 experts from diverse disciplines—including physical education, adolescent health promotion, and public health—were invited, and all completed both the first and second rounds of the Delphi survey. The panel covered multiple regions of China as well as one overseas expert, thereby enhancing representativeness. A summary of the expert information is presented in Table 1 (with detailed demographic data provided in Appendix Table 1). Years of professional experience were calculated up to December 31, 2024—prior to the formal Delphi rounds conducted between January and March 2025—ensuring consistency across all expert data.

**Table 1:** Characteristics of the experts (*n* = 17).

Demographic	Category	*n*(%)/mean
Gender	Male	13 (76%)
	Female	4 (24%)
Years of professional experience^a^	Average	21.2
	Range	5–35
Education background	Bachelor	1 (6%)
	Master	3 (18%)
	PhD	13 (76%)
Professional job title^b^	Professor	8 (47%)
	Associate professor	9 (53%)
Area of expertise	Physical education	6 (35%)
	Adolescent health promotion	3 (18%)
	Physical activity and health	7 (41%)
	Physical exercise and psychosomatic health	1 (6%)

a Years of professional experience were calculated up to December 31, 2024.

b All professional job titles were standardized based on the commonly used academic rank system in China: teaching assistant, assistant professor, associate professor, and professor.

### 2.4 Telephone interview

As a well-established method, telephone interviews are widely applied in the field of sociology and are frequently used in Delphi studies (13, 14). Therefore, prior to initiating the Delphi survey, telephone interviews were conducted with all 17 selected experts between January and February 2025. The purpose of these interviews was to gather expert opinions on the importance, comprehensiveness, and relevance of the proposed framework, and to revise the initial theoretical structure and indicator pool accordingly.

Drawing on both the systematic literature review and the expert interviews, an initial pool of 61 candidate indicators was generated. Overlapping or redundant items were merged, and each indicator was categorized into one of the five proposed domains: school leadership, family involvement, community support, government support, and societal collaboration. This process produced a preliminary framework comprising 5 structural components, 17 primary indicators, and 61 secondary indicators, which subsequently served as the basis for developing the first-round Delphi questionnaire.

### 2.5 Delphi study

The Delphi method is a research approach particularly suited to addressing practical problems that require expert input (15). In this study, we adopted a modified Delphi approach, which differed from the classical Delphi in three main ways: (1) only two iterative rounds were conducted instead of three or more; (2) a preliminary interview phase was integrated to generate candidate items; and (3) pre-specified statistical thresholds were applied to determine item retention. These modifications were designed to reduce participant fatigue while ensuring methodological rigor and practical feasibility.

The survey was conducted entirely online using an iterative mixed-methods design to achieve expert consensus. To minimize potential biases—especially social desirability bias—and to encourage independent judgment, all responses were anonymized. Each expert received a personalized survey link, while identifying information was stored separately from response data. Only aggregated statistics were shared with the panel, and qualitative comments were de-identified prior to circulation to ensure confidentiality. After each round, the research team synthesized the quantitative ratings and qualitative feedback, revised the questionnaire accordingly, and provided participants with a summary report including consensus statistics and thematically coded comments to support informed re-evaluation. Electronic informed consent was obtained from all experts prior to participation.

### 2.6 Two-round Delphi survey

Two rounds of the Delphi survey were conducted to balance the pursuit of expert consensus with the risk of participant fatigue and declining response rates—challenges commonly encountered in multi-round Delphi studies (16). Previous research has shown that two rounds are generally sufficient to stabilize expert opinions, particularly when the initial framework is grounded in empirical evidence and informed by preliminary interviews (13, 14). The Delphi process was carried out online between March and May 2025.

The first-round questionnaire was developed based on a comprehensive literature review and insights from preliminary expert interviews. In Round 1, 17 experts were invited to complete the survey within two weeks, with a reminder sent after 10 days if necessary. Experts were asked to evaluate the framework’s components, primary indicators, and secondary indicators using a five-point Likert scale (1 = strongly disagree, 2 = disagree, 3 = neutral, 4 = agree, 5 = strongly agree). They were also encouraged to provide open-ended suggestions regarding modification, retention, or deletion of items.

Based on the Round 1 results, the research team conducted thematic analysis of the qualitative feedback from open-ended responses and integrated it with consensus rates to develop the Round 2 questionnaire. This ensured that iterative decisions were guided not only by statistical thresholds but also by expert reasoning and contextual insights.

Before completing the Round 2 questionnaire, each expert received a structured feedback report containing their own Round 1 ratings alongside descriptive statistics (mean, SD, CV, % ≥ 4, FF) for each indicator, as well as an anonymized thematic synthesis of qualitative comments. This detailed feedback enabled experts to re-evaluate revised items in light of both quantitative evidence and the collective perspectives of the panel.

### 2.7 Consensus

To determine expert consensus across the two Delphi rounds, explicit criteria were applied at each stage. In the first round, consensus was defined as at least 80% of experts rating an item as “4” (agree) or “5” (strongly agree), a threshold commonly adopted in Delphi research (15). Items meeting this threshold were retained directly.

In the second round, three complementary criteria were used to provide a more stringent and stable measure of consensus: (1) mean score ≥4.0; (2) coefficient of variation (CV) ≤ 0.25; and (3) frequency of full scores (FF, proportion of experts rating “5”) ≥ 0.50. Items meeting all three criteria were considered to have reached consensus.

In rare cases where an item narrowly failed to meet one criterion—typically the CV threshold—but was strongly endorsed by qualitative feedback and deemed theoretically essential by the research team, it was retained with justification.

To ensure transparency, Appendix Table 2 presents per-item statistics for first rounds (mean, SD, CV, FF, % ≥ 4), allowing replication and verification of the consensus process.

### 2.8 Analytic hierarchy process

To determine the relative importance of elements within the framework, the AHP was applied after completion of the second Delphi round. Experts were asked to conduct pairwise comparisons using a structured judgment matrix across three levels: (1) components, (2) primary indicators under each component, and (3) secondary indicators under each primary indicator.

All pairwise comparisons were quantified using the 1–9 Saaty scale to capture experts’ judgments. Each comparison assessed the relative importance of two elements with respect to the higher-level criterion. These pairwise judgments were used to construct reciprocal matrices, forming the basis for calculating the relative weights of components, primary indicators, and secondary indicators.

Group judgments were aggregated using the arithmetic mean method, which was chosen for its simplicity, transparency, and frequent application in education and health-related AHP studies, ensuring that the aggregated matrices preserved the intuitive interpretation of expert inputs.

To ensure logical consistency in expert evaluations, a consistency ratio (CR) was calculated for each matrix. A CR value ≤ 0.1 was considered acceptable. All CR values for aggregated matrices are reported in Appendix Table 4. For individual cases with CR > 0.10, experts were contacted to revise their responses. Matrices that remained inconsistent after revision were excluded. Detailed counts are provided in Appendix Table 4.

Representative aggregated pairwise comparison matrices are also provided in Appendix Tables 5–10, along with examples of individual matrices, to enhance transparency and replicability.

All AHP calculations—including construction of judgment matrices, consistency ratio computations, and derivation of final weights—were performed using YAAHP software (version 12.6).

### 2.9 Data analysis

Descriptive statistics of expert characteristics (e.g., frequencies, percentages, means, and standard deviations), consensus rates, and item rating distributions were analyzed using IBM SPSS Version 27.0.

The AHP was employed to determine the specific weights of components, primary indicators, and secondary indicators within the framework. Pairwise comparison data provided by experts were analyzed using YAAHP software (Yuanjue Decision Software Technology Co., Ltd., Taiyuan, China; version 12.6).

## 3 Results

### 3.1 Theoretical model of physical activity promotion for children and adolescents

To enable a clearer and more focused examination of PA, this study—drawing on the existing literature—defines PA as “any bodily movement produced by skeletal muscle contraction that results in energy expenditure,” encompassing leisure-time exercise, community activities, household tasks, active transportation, and work-related activities (17).

Developing a framework for PA promotion requires targeted actions that take into account the factors influencing PA. McLeroy, who first introduced the socio-ecological perspective into health behavior research, emphasized that individual, interpersonal, institutional, community, and public policy factors play important roles in shaping health behaviors (18). The Social Ecological Model, as an evolving theoretical framework, has been empirically validated in numerous international studies and is widely regarded as an effective framework for systematically analyzing factors influencing PA (19).

Furthermore, a systematic analysis of PA promotion strategies included in 28 international guidelines for children and adolescents revealed that these strategies emphasize multi-sectoral collaboration, involving schools, families, healthcare systems, communities, sports organizations, governments, urban planning, transportation, environmental management, and mass media. Among these, schools were most frequently highlighted, followed by families, healthcare, and communities.

**Figure 1:**
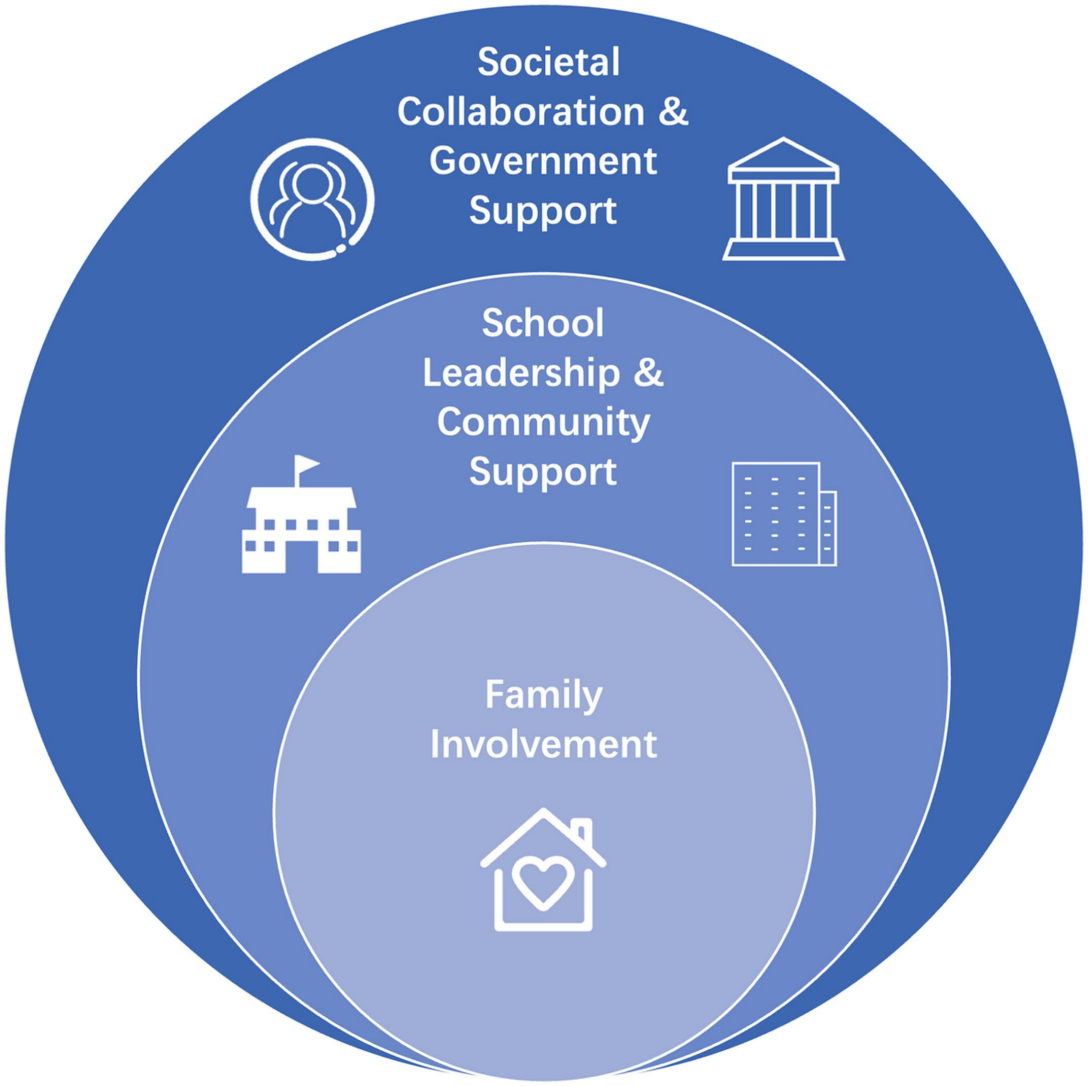
Five core components of physical activity promotion. The framework is adapted from McLeroy’s social ecological model, illustrating how macro-level factors (societal collaboration and government support) provide the overarching environment that shapes meso-level contexts (school leadership and community support), which in turn influence micro-level environments such as family involvement.

Based on this evidence, the present study developed a preliminary theoretical model for promoting PA among children and adolescents, comprising five key dimensions: school leadership, family involvement, community support, government support, and societal collaboration. As shown in Figure 1, these five components are organized hierarchically based on the Social Ecological Model (18), with societal collaboration and government support at the macro level, schools and communities at the meso level, and families at the micro level. This nested structure highlights how higher-level determinants provide enabling conditions for lower-level actors.

### 3.2 Development of the initial framework

The overall structure of this study was grounded in systems theory, dividing the framework into three interrelated levels. While elements across different levels were interconnected, those within the same level were designed to be independent yet complementary, forming a coherent framework system (see Table 1).

The initial set of dimensions was informed by socio-ecological theory, which delineates five domains: schools, families, communities, government, and society. The identification of primary and secondary indicators was further informed by theories at multiple levels: individual-level theories such as the Health Belief Model and the Transtheoretical Model; interpersonal-level theories such as Social Cognitive Theory (20), Social Network Theory, and Social Support Theory (21); and group-level theories such as Communication Theory and Community Mobilization Theory.

At the same time, relevant research on models and strategies for promoting PA among children and adolescents was referenced to ensure that the framework developed in this study would be systematic, comprehensive, and scientifically grounded. These references included, but were not limited to, the WHO Global Action Plan on Physical Activity 2018–2030: More Active People for a Healthier World (6), the WHO Guidelines on Physical Activity and Sedentary Behavior (1), the Guidelines on Physical Activity for Chinese Children and Adolescents (11), and the 2018 Physical Activity Guidelines Advisory Committee Scientific Report from the United States (7).

Drawing on literature, international experience, the current situation of PA promotion among Chinese children and adolescents, and practical lessons, the research team conducted brainstorming sessions. As a result, an initial framework was developed, consisting of five dimensions, 17 primary indicators, and 61 secondary indicators. The complete preliminary indicator pool is presented in Table 2.

**Table 2:** Framework of indicators for promoting physical activity among children and adolescents (preliminary draft).

Dimension (5)	Primary indicator (17)	Secondary indicator (61)
1 School leadership	1.1 Classroom instruction	1.1.1 PE and health classes
		1.1.2 Micro-activities in academic classes
		1.1.3 Cultural and sports integrated curriculum
	1.2 Extracurricular activities	1.2.1 Morning exercises
		1.2.2 Breaktime exercises
		1.2.3 Sports training
		1.2.4 Sports competitions
		1.2.5 Sports interest groups/clubs/associations
		1.2.6 Sports festivals/sports meets
		1.2.7 Parent–school collaborative sports activities
	1.3 Teacher workforce	1.3.1 Teacher quantity
		1.3.2 Teacher quality
	1.4 Facilities & equipment	1.4.1 Facility provision
		1.4.2 Facility safety
		1.4.3 Facility accessibility
		1.4.4 Facility maintenance
	1.5 School sports culture	1.5.1 Sports activity atmosphere
		1.5.2 Sports information and publicity
		1.5.3 Teacher–student sports awareness
	1.6 Organizational management	1.6.1 Organizational structure
		1.6.2 Work systems and plans
		1.6.3 Financial support
		1.6.4 Monitoring and feedback
		1.6.5 Evaluation and incentives
2 Family involvement	2.1 Parental attitudes	2.1.1 Verbal encouragement
		2.1.2 Active concern
		2.1.3 Communication of benefits
	2.2 Parental behaviors	2.2.1 Parents’ own participation
		2.2.2 Joint parent–child participation
		2.2.3 Parents observing children’s participation
	2.3 Family atmosphere	2.3.1 Financial support for sports
		2.3.2 Space provision for activities
		2.3.3 Limiting sedentary behaviors
3 Community support	3.1 Sports facilities	3.1.1 Public sports facilities
		3.1.2 Commercial fitness clubs
		3.1.3 Community children’s activity centers
	3.2 Sports environment	3.2.1 Community open space utilization
		3.2.2 Community safety
		3.2.3 Daily PA signage
		3.2.4 Walkability of community roads
		3.2.5 Accessibility of fitness venues
	3.3 Health services	3.3.1 Health education and counseling
		3.3.2 Health skills guidance/training
		3.3.3 Organization of sports activities
		3.3.4 Health information dissemination
	3.4 Organizational management	3.4.1 Community sports organizations
		3.4.2 Community sports regulations
4 Government support	4.1 Institutional guarantees	4.1.1 Policy support
		4.1.2 Legal constraints
		4.1.3 Financial support
		4.1.4 Supervision and evaluation
		4.1.5 Information disclosure
	4.2 Environmental development	4.2.1 Cultural environment
		4.2.2 Ecological environment
5. Societal collaboration	5.1 Incentive mobilization	5.1.1 Public awareness
		5.1.2 Group action support
	5.2 Resource integration	5.2.1 Facility and equipment resources
		5.2.2 Human resources
		5.2.3 Financial resources
		5.2.4 Information resources
		5.2.5 Technological resources

### 3.3 Results of the first round of the Delphi survey

In the first round of the Delphi process, questionnaires were distributed to 17 experts, and 17 valid responses were received. Feedback and corresponding revisions on the five dimensions, 17 primary indicators, and 61 secondary indicators are summarized in Table 3.

**Table 3:** Statistical results of the first-round expert consultation.

Indicator	Retained (*n*)	Approval rate	Expert feedback
1 School leadership	17	100.00%	—
2 Family involvement	17	100.00%	—
3 Community support	16	94.12%	—
4 Government support	15	88.24%	Suggested merger with “E. societal collaboration”
5 Societal collaboration	16	94.12%	—
1.1 Classroom instruction	17	100.00%	—
1.2 Extracurricular activities	17	100.00%	—
1.3 Teacher workforce	17	100.00%	—
1.4 Facilities & equipment	16	94.12%	—
1.5 School sports culture	16	94.12%	—
1.6 Organizational management	17	100.00%	Suggested to split out “A7 institutional guarantee”
2.1 Parental attitudes	17	100.00%	—
2.2 Parental behaviors	17	100.00%	—
2.3 Family atmosphere	17	100.00%	—
3.1 Sports facilities	17	100.00%	—
3.2 Sports environment	15	88.24%	Suggested revision
3.3 Health services	15	88.24%	—
3.4 Organizational management	16	94.12%	—
4.1 Institutional guarantee	16	94.12%	—
4.2 Environmental development	16	94.12%	—
5.1 Incentive mobilization	16	94.12%	—
5.2 Resource integration	15	88.24%	—
1.1.1 PE and health classes	17	100.00%	—
1.1.2 Micro-activities in academic classes	13	76.47%	—
1.1.3 Cultural and sports integrated curriculum	14	82.35%	Suggested revision
1.2.1 Morning exercises	16	94.12%	Suggested revision
1.2.2 Breaktime exercises	16	94.12%	Suggested revision
1.2.3 Sports training	15	88.24%	Suggested revision
1.2.4 Sports competitions	17	100.00%	—
1.2.5 Sports interest groups/clubs/associations	17	100.00%	—
1.2.6 Sports festivals/sports meets	16	94.12%	—
1.2.7 Parent–school collaborative sports activities	16	94.12%	Suggested revision
1.3.1 Teacher quantity	17	100.00%	—
1.3.2 Teacher quality	17	100.00%	—
1.4.1 Facility provision	17	100.00%	—
1.4.2 Facility safety	16	94.12%	—
1.4.3 Facility accessibility	17	100.00%	—
1.4.4 Facility maintenance	16	94.12%	—
1.5.1 Sports activity atmosphere	17	100.00%	—
1.5.2 Sports information and publicity	16	94.12%	—
1.5.3 Teacher–student sports awareness	15	88.24%	—
1.6.1 Organizational structure	16	94.12%	Suggested revision
1.6.2 Work systems and plans	16	94.12%	—
1.6.3 Financial support	17	100.00%	Reassigned to “A7 institutional guarantee”
1.6.4 Monitoring and feedback	17	100.00%	Reassigned to “A7 institutional guarantee”
1.6.5 Evaluation and incentives	16	94.12%	—
2.1.1 Verbal encouragement	17	100.00%	—
2.1.2 Active concern	16	94.12%	—
2.1.3 Communication of benefits	16	94.12%	Suggested addition of “time support”
2.2.1 Parents’ own participation	16	94.12%	—
2.2.2 Joint parent–child participation	17	100.00%	—
2.2.3 Parents observing children’s participation	15	88.24%	Suggested revision
2.3.1 Financial support for sports	17	100.00%	—
2.3.2 Space provision for activities	15	88.24%	—
2.3.3 Limiting sedentary behaviors	15	88.24%	—
3.1.1 Public sports facilities	17	100.00%	—
3.1.2 Commercial fitness clubs	12	70.59%	Suggested revision
3.1.3 Community children’s activity centers	17	100.00%	—
3.2.1 Community open space utilization	14	82.35%	Suggested revision
3.2.2 Community safety	14	82.35%	—
3.2.3 Daily PA signage	11	64.71%	Suggested revision
3.2.4 Walkability of community roads	15	88.23%	Suggested revision
3.2.5 Accessibility of fitness venues	17	100.00%	Suggested revision
3.3.1 Health education and counseling	13	76.47%	—
3.3.2 Health skills guidance/training	15	88.24%	—
3.3.3 Organization of sports activities	15	88.24%	—
3.3.4 Health information dissemination	12	70.59%	—
3.4.1 Community sports organizations	15	88.24%	Suggested revision
3.4.2 Community sports regulations	13	76.47%	Suggested revision
4.1.1 Policy support	17	100.00%	—
4.1.2 Legal constraints	17	100.00%	—
4.1.3 Financial support	17	100.00%	—
4.1.4 Supervision and evaluation	16	94.12%	—
4.1.5 Information disclosure	15	88.24%	—
4.2.1 Cultural environment	16	94.12%	—
4.2.2 Ecological environment	16	94.12%	—
5.1.1 Public awareness	17	100.00%	—
5.1.2 Group action support	17	100.00%	—
5.2.1 Facility & equipment resources	17	100.00%	—
5.2.2 Human resources	16	94.12%	—
5.2.3 Financial resources	16	94.12%	—
5.2.4 Information resources	15	88.24%	—
5.2.5 Technological resources	15	88.24%	—

For the five dimensions, the consensus rate among experts was very high. Statistical results showed an average approval rate of 95.29%, with only the fourth dimension, government support, receiving a consensus rate of 88.24%. Several experts suggested that government support and societal collaboration represented similar attributes and should be merged into a single dimension. Taking this into account, the research team combined these two dimensions into a new dimension, societal collaboration, resulting in four overarching dimensions.

For the 17 primary indicators, the average approval rate was 95.85%. Most experts focused on whether the vertical logic between dimensions and primary indicators was coherent and whether the horizontal relationships between indicators were clearly defined. Based on this feedback, the research team carefully reviewed each indicator and made revisions accordingly. Experts suggested that the concept of sports environment was inaccurate, and that built environment would be more appropriate. Therefore, 3.2 Sports Environment was revised to 3.2 Built Environment. Additionally, some experts recommended splitting 1.6 Organizational Management into two indicators: 1.6 Organizational Management and 1.7 Institutional Guarantee, with 1.6.3 Financial Support and 1.6.4 Monitoring and Feedback reassigned as sub-indicators under the new 1.7 Institutional Guarantee. After thorough discussion with other experts, this recommendation was adopted. Consequently, the number of primary indicators increased to 18 after the first round.

Regarding the 61 secondary indicators, expert feedback mainly focused on internal logic, consistency with parent indicators, and clarity of expression. Many practitioners recommended making the indicators more specific to enhance their practical applicability. In response, the research team refined the clarity and relevance of each indicator. Specifically, one secondary indicator, 2.1.4 Time Support, was added, and 14 secondary indicators were modified. For example, 1.1.3 Cultural and Sports Integrated Curriculum was revised to 1.1.3 Interdisciplinary Integrated Physical Education Curriculum; 1.2.1 Morning Exercises to 1.2.1 Morning Physical Activities; 1.2.2 Breaktime Exercises to 1.2.2 Extended Recess Physical Activities; and 1.2.3 Sports Training to 1.2.3 Extracurricular Training. These revisions enhanced the professionalism of terminology and the clarity of indicators.

For the six indicators that did not meet the 80% consensus threshold, the research team carefully reviewed the expert suggestions and conducted further analyses and discussions with relevant experts. Ultimately, the conceptual importance of these indicators justified their retention, with minor wording refinements made to address concerns. Table 3 presents the statistical summary of expert feedback after the first round.

### 3.4 Results of the second round of the Delphi survey

Building on the results of the first round, a Version 2.0 framework was developed. This version included four dimensions—school leadership, family involvement, community support, and societal collaboration—along with 18 primary indicators and 62 secondary indicators (Table 4). In the second Delphi round, the revised framework was distributed to the same 17 experts, all of whom completed the survey. Feedback and results are summarized in Table 5.

**Table 4:** Indicator system of the second-round expert consultation.

Dimension (4)	Primary indicator (18)	Secondary indicator (62)
1 School leadership	1.1 Classroom instruction	1.1.1 PE and health classes
		1.1.2 Micro-activities in academic classes
		1.1.3 Interdisciplinary integrated PE curriculum
	1.2 Extracurricular activities	1.2.1 Morning physical activities
		1.2.2 Extended recess physical activities
		1.2.3 Extracurricular training
		1.2.4 Sports competitions
		1.2.5 Sports interest groups/clubs/associations
		1.2.6 Sports festivals/sports meets
		1.2.7 Parent–school collaborative activities
	1.3 Teacher workforce	1.3.1 Teacher quantity
		1.3.2 Teacher quality
	1.4 Facilities & equipment	1.4.1 Facility provision
		1.4.2 Facility safety
		1.4.3 Facility accessibility
		1.4.4 Facility maintenance
	1.5 School sports culture	1.5.1 Sports activity atmosphere
		1.5.2 Sports information and publicity
		1.5.3 Teacher–student sports awareness
	1.6 Organizational management	1.6.1 Working group
		1.6.2 Work systems and plans
		1.6.3 Evaluation and incentives
	1.7 Institutional guarantee	1.7.1 Financial support
		1.7.2 Monitoring and feedback
2 Family involvement	2.1 Parental attitudes	2.1.1 Verbal encouragement
		2.1.2 Active concern
		2.1.3 Communication of benefits
		2.1.4 Time support
	2.2 Parental behaviors	2.2.1 Parents’ own participation
		2.2.2 Joint parent–child participation
		2.2.3 Parents guiding children’s participation
	2.3 Family atmosphere	2.3.1 Financial support for sports
		2.3.2 Space provision for activities
		2.3.3 Limiting sedentary behaviors
3 Community support	3.1 Sports facilities	3.1.1 Public sports facilities
		3.1.2 Sports and fitness clubs
		3.1.3 Community children’s activity centers
	3.2 Built environment	3.2.1 Community space utilization
		3.2.2 Community safety
		3.2.3 Motivational signage for PA
		3.2.4 Community walkability
		3.2.5 Accessibility of community sports venues
	3.3 Health services	3.3.1 Health education and counseling
		3.3.2 Health skills guidance/training
		3.3.3 Organization of sports activities
		3.3.4 Health information dissemination
	3.4 Organizational management	3.4.1 Community sports institutions
		3.4.2 Community sports support system
4 Societal collaboration	4.1 Institutional guarantee	4.1.1 Policy support
		4.1.2 Legal constraints
		4.1.3 Financial support
		4.1.4 Supervision and evaluation
		4.1.5 Information disclosure
	4.2 Environmental development	4.2.1 Cultural environment
		4.2.2 Ecological environment
	4.3 Incentive mobilization	4.3.1 Public awareness
		4.3.2 Group action support
	4.4 Resource integration	4.4.1 Facility & equipment resources
		4.4.2 Human resources
		4.4.3 Financial resources
		4.4.4 Information resources
		4.4.5 Technological resources

**Table 5:** Summary of expert consultation results in the second round.

Indicator name	*M*	SD	CV	FF	M ≥ M-2 × SD	CV ≤ CV+2 × SD	FF ≥ FF-2 × SD
1 School leadership	5	0	0	1	5	0	1
2 Family involvement	5	0	0	1	5	0	1
3 Community support	4.1176	0.5	0.1214	0.288	3.12	1.12	−0.71
4 Societal collaboration	4.2108	0.6251	0.1213	0.404	2.96	1.37	−0.85
1.1 Classroom instruction	5	0	0	1	5	0	1
1.2 Extracurricular activities	4.9412	0.25	0.0506	0.938	4.44	0.55	0.44
1.3 Teacher workforce	4.8824	0.3416	0.07	0.875	4.2	0.75	0.19
1.4 Facilities & equipment	4.5882	0.6292	0.1371	0.625	3.33	1.4	−0.63
1.5 School sports culture	4.4118	0.6191	0.1403	0.438	3.17	1.38	−0.8
1.6 Organizational management	4.5294	0.5164	0.114	0.5	3.5	1.15	−0.53
1.7 Institutional guarantee	4.1318	0.3802	0.1103	0.664	3.37	0.87	−0.1
2.1 Parental attitude	4.9412	0.25	0.0506	0.938	4.44	0.55	0.44
2.2 Parental behavior	4.9412	0.25	0.0506	0.938	4.44	0.55	0.44
2.3 Family atmosphere	4.3529	0.6021	0.1383	0.375	3.15	1.34	−0.83
3.1 Sports facilities	4.5882	0.5123	0.1117	0.563	3.56	1.14	−0.46
3.2 Built environment	3.8824	0.7188	0.1851	0.188	2.44	1.62	−1.25
3.3 Health services	3.8235	0.9106	0.2382	0.25	2	2.06	−1.57
3.4 Organizational management	3.8824	0.6191	0.1595	0.125	2.64	1.4	−1.11
4.1 Institutional guarantee	4.5294	0.6292	0.1389	0.625	3.27	1.4	−0.63
4.2 Environmental development	4.4118	0.6292	0.1426	0.5	3.15	1.4	−0.76
4.3 Incentive mobilization	3.5882	0.6325	0.2581	0.188	2.32	1.52	−1.08
4.4 Resource integration	4.3529	0.6021	0.1383	0.375	3.15	1.34	−0.83
1.1.1 PE and health classes	5	0	0	1	5	0	1
1.1.2 Micro-activities in academic classes	3.7647	0.9309	0.2473	0.25	1.9	2.11	−1.61
1.1.3 Interdisciplinary integrated PE curriculum	4.0588	0.7719	0.1902	0.313	2.51	1.73	−1.23
1.2.1 Morning physical activities	4.1765	0.6551	0.1569	0.313	2.87	1.47	−1
1.2.2 Extended recess physical activities	4.7647	0.4472	0.0939	0.75	3.87	0.99	−0.14
1.2.3 Extracurricular training	4.1176	0.5	0.1214	0.188	3.12	1.12	−0.81
1.2.4 Sports competitions	4.5294	0.5164	0.114	0.5	3.5	1.15	−0.53
1.2.5 Sports interest groups/clubs/associations	4.8235	0.4031	0.0836	0.813	4.02	0.89	0.01
1.2.6 Sports festivals/sports meets	4.5294	0.5164	0.114	0.5	3.5	1.15	−0.53
1.2.7 Parent–school collaborative activities	4.3529	0.6191	0.1422	0.438	3.11	1.38	−0.8
1.3.1 Teacher quantity	4.4118	0.6292	0.1426	0.5	3.15	1.4	−0.76
1.3.2 Teacher quality	4.9412	0.25	0.0506	0.938	4.44	0.55	0.44
1.4.1 Facility provision	4.7647	0.4472	0.0939	0.75	3.87	0.99	−0.14
1.4.2 Facility safety	4.7647	0.4472	0.0939	0.75	3.87	0.99	−0.14
1.4.3 Facility accessibility	4.5882	0.6292	0.1371	0.625	3.33	1.4	−0.63
1.4.4 Facility maintenance	4.2353	0.5774	0.1363	0.188	3.08	1.29	−0.97
1.5.1 Sports activity atmosphere	4.7647	0.4472	0.0939	0.75	3.87	0.99	−0.14
1.5.2 Sports information and publicity	4.1176	0.8851	0.2149	0.375	2.35	1.99	−1.4
1.5.3 Teacher–student sports awareness	4.6471	0.6191	0.1332	0.688	3.41	1.37	−0.55
1.6.1 Working group	4.4706	0.5123	0.1146	0.438	3.45	1.14	−0.59
1.6.2 Work systems and plans	4.4706	0.6292	0.1407	0.5	3.21	1.4	−0.76
1.6.3 Evaluation and incentives	4.8235	0.4031	0.0836	0.813	4.02	0.89	0.01
1.7.1 Financial support	4.5882	0.5123	0.1117	0.563	3.56	1.14	−0.46
1.7.2 Monitoring and feedback	4.5294	0.6325	0.1396	0.563	3.26	1.4	−0.7
2.1.1 Verbal encouragement	4.5882	0.5123	0.1117	0.563	3.56	1.14	−0.46
2.1.2 Active concern	4.6471	0.5	0.1076	0.625	3.65	1.11	−0.38
2.1.3 Communication of benefits	4.3529	0.4787	0.11	0.313	3.4	1.07	−0.64
2.1.4 Time support	4.7647	0.4472	0.0939	0.75	3.87	0.99	−0.14
2.2.1 Parents’ own participation	4.4118	0.6191	0.1403	0.438	3.17	1.38	−0.8
2.2.2 Joint parent–child participation	4.8824	0.3416	0.07	0.875	4.2	0.75	0.19
2.2.3 Parents guiding children’s participation	4.0588	0.8165	0.2012	0.313	2.43	1.83	−1.32
2.3.1 Financial support for sports	4.7059	0.4787	0.1017	0.688	3.75	1.06	−0.27
2.3.2 Space provision for activities	4	0.6325	0.1581	0.313	2.74	1.42	−0.95
2.3.3 Limiting sedentary behaviors	4.2353	0.6831	0.1613	0.375	2.87	1.53	−0.99
3.1.1 Public sports facilities	4.5882	0.5123	0.1117	0.563	3.56	1.14	−0.46
3.1.2 Sports and fitness clubs	3.5882	0.7274	0.2027	0.125	2.13	1.66	−1.33
3.1.3 Community children’s activity centers	4.3529	0.5	0.1149	0.375	3.35	1.11	−0.62
3.2.1 Community space utilization	3.8235	0.9811	0.2566	0.35	1.86	2.22	−1.61
3.2.2 Community safety	4.1765	0.8342	0.1997	0.375	2.51	1.87	−1.29
3.2.3 Motivational signage for PA	3.7647	1.0646	0.2828	0.313	1.64	2.41	−1.82
3.2.4 Community walkability	3.8235	0.8342	0.2182	0.318	2.16	1.89	−1.35
3.2.5 Accessibility of community sports venues	4.4706	0.5164	0.1155	0.5	3.44	1.15	−0.53
3.3.1 Health education and counseling	3.9412	0.7188	0.1824	0.375	2.5	1.62	−1.06
3.3.2 Health skills guidance/training	4.2941	0.6831	0.1591	0.375	2.93	1.53	−0.99
3.3.3 Organization of sports activities	4.4118	0.5123	0.1161	0.438	3.39	1.14	−0.59
3.3.4 Health information dissemination	3.8824	0.7188	0.1851	0.318	2.44	1.62	−1.12
3.4.1 Community sports institutions	4.1176	0.5	0.1214	0.318	3.12	1.12	−0.68
3.4.2 Community sports support system	3.7333	0.9106	0.2439	0.375	1.91	2.07	−1.45
4.1.1 Policy support	4.6667	0.5	0.1071	0.625	3.67	1.11	−0.38
4.1.2 Legal constraints	4.4667	0.6325	0.1416	0.563	3.2	1.41	−0.7
4.1.3 Financial support	4.8	0.5439	0.1133	0.875	3.71	1.2	−0.21
4.1.4 Supervision and evaluation	4.6	0.5	0.1087	0.625	3.6	1.11	−0.38
4.1.5 Information disclosure	3.9333	0.5737	0.1459	0.313	2.79	1.29	−0.83
4.2.1 Cultural environment	4.1333	0.75	0.1815	0.313	2.63	1.68	−1.19
4.2.2 Ecological environment	4	0.7719	0.193	0.438	2.46	1.74	−1.11
4.3.1 Public awareness	4.4667	0.6325	0.1416	0.313	3.2	1.41	−0.95
4.3.2 Group action support	4.3333	0.6191	0.1429	0.313	3.1	1.38	−0.93
4.4.1 Facility & equipment resources	4.5333	0.5123	0.113	0.563	3.51	1.14	−0.46
4.4.2 Human resources	4.2667	0.4787	0.1122	0.313	3.31	1.07	−0.64
4.4.3 Financial resources	4.5333	0.6292	0.1388	0.625	3.27	1.4	−0.63
4.4.4 Information resources	3.8667	0.5737	0.1484	0.313	2.72	1.3	−0.83
4.4.5 Technological resources	4.0667	0.6191	0.1522	0.25	2.83	1.39	−0.99

For the dimension indicators, all four dimensions received mean importance scores (M) above the threshold of 3.5, indicating strong expert recognition of their relevance. Specifically, school leadership (M = 5.00) and family involvement (M = 5.00) scored the highest, followed by community support (M = 4.1176) and societal collaboration (M = 4.2108). All CV were below 0.25, reflecting low variability in expert assessments. The FF for community was 28.75%, close to the 30% benchmark. Overall, most indicators met all three predefined screening criteria: (1) M ≥ M – 2SD, (2) FF ≥ FF – 2SD, and (3) CV ≤ CV + 2SD. Qualitative feedback supported these quantitative findings. Based on this combined analysis, four final dimensions were confirmed: school leadership, family involvement, community support, and societal collaboration.

For the primary indicators, all 18 had mean importance scores greater than 3.5 (overall mean = 4.4740). With the exception of 4.3 Incentive Mobilization, all CV values were below 0.25, indicating a high degree of expert consensus. Four indicators had FF values below 30%: 3.2 Built Environment (18.75%), 3.3 Health Services (25.00%), 3.4 Organizational Management (12.5%), and 4.3 Incentive Mobilization (18.75%). After further consultation with experts, 3.2 Built Environment, 3.3 Health Services, and 3.4 Organizational Management were retained, while 4.3 Incentive Mobilization was deleted. Consequently, 17 primary indicators were included in the final framework.

For the secondary indicators, all 62 indicators had mean importance scores (M) above 3.5, ranging from 3.5882 to 5.00. Five indicators had FF values below 30%: 1.1.2 Micro-activities in Academic Classes (25.00%), 1.2.3 Extracurricular Training (18.75%), 1.4.4 Facility Maintenance (15.75%), 3.1.2 Sports and Fitness Clubs (12.50%), and 4.4.5 Technological Resources (25.00%). Based on in-depth discussions combining expert feedback with existing evidence, the following decisions were made:

- 1.1.2 Micro-activities in Academic Classes was retained, as research supports the health benefits of fragmented activity and its positive role in learning contexts.
- 1.2.3 Extracurricular Training was merged with 1.2.4 Extracurricular Competitions into a new indicator, 1.2.3 After-school Training/Competitions.
- 1.4.4 Facility Maintenance was deleted due to overlap with 1.4.2 Facility Safety.
- 3.1.2 Sports and Fitness Clubs was retained but rephrased as Youth Sports Clubs to better reflect its relevance.
- 4.4.5 Technological Resources was retained, given its emphasis on the role of artificial intelligence in PA promotion, despite an FF slightly below 30%.

Two indicators—3.2.1 Community Space Utilization and 3.2.3 Motivational Signage for Physical Activity—had CV values above 0.25 (0.2566 and 0.2828, respectively). After further discussion, experts agreed these represented areas requiring improvement in China’s PA promotion strategies and thus should be retained.

Because the primary indicator 4.3 Incentive Mobilization was deleted, its two corresponding secondary indicators were also removed. Based on the combined quantitative and qualitative analyses, the following revisions were made: three secondary indicators deleted, two merged into one, and one rephrased. Ultimately, 58 secondary indicators were retained.

**Figure 2:**
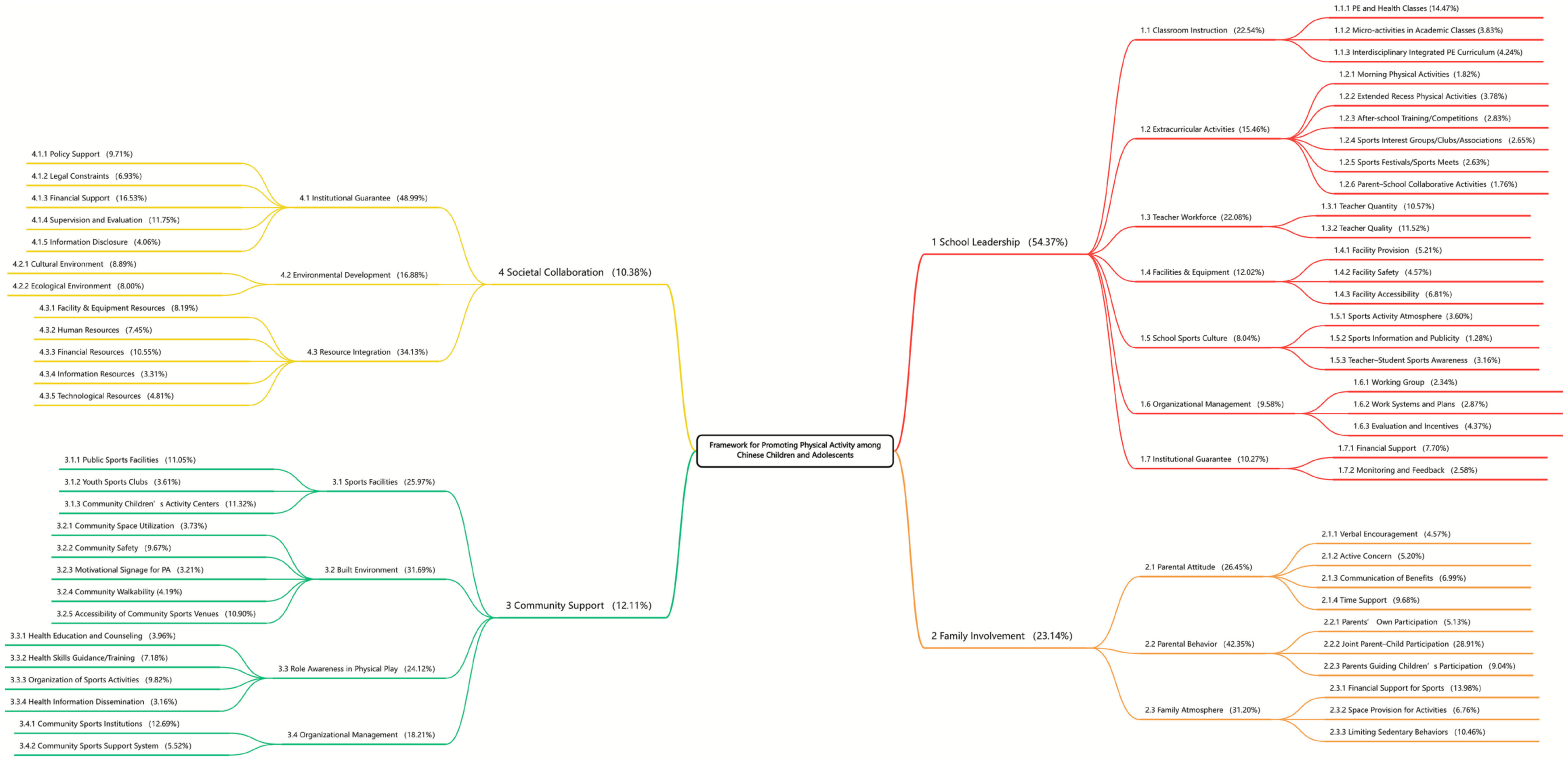
Framework for promoting physical activity among chinese children and adolescents.

Following the second round of analysis and revisions, the final framework for promoting PA among children and adolescents was established, consisting of four dimensions, 17 primary indicators, and 58 secondary indicators (Figure 2; Appendix Table 3).

### 3.5 Weights of indicators in the framework

The results of the AHP revealed the following weight distribution across the four core dimensions of the framework: school leadership carried the highest weight at 54.37%, followed by family involvement at 23.14%. The weights for community support and societal collaboration were relatively lower, at 12.11 and 10.38%, respectively.

Overall, these findings suggest that while schools, families, communities, and society must work together to promote PA among children and adolescents, schools play the most critical role. Therefore, when designing interventions for PA promotion, schools should serve as the primary platform, guiding family engagement and mobilizing community and societal resources to collectively enhance PA levels in children and adolescents.

The detailed weights for all dimensions, primary indicators, and secondary indicators are presented in Figure 2 and Appendix Table 3.

## 4 Discussion

In light of the current status of PA promotion among Chinese children and adolescents and within the broader context of national policy, this study represents the first systematic attempt to construct a comprehensive framework for PA promotion in this population. The resulting framework encompasses four core dimensions—school leadership, family involvement, community support, and societal collaboration—alongside 17 primary indicators and 58 secondary indicators, each assigned a corresponding weight coefficient.

### 4.1 School leadership

Schools are the most direct and widespread institutions for delivering health education activities. The expert survey results indicated that schools received the highest weight coefficient and the highest full-score frequency, reflecting a strong consensus among experts that schools hold a central position in promoting PA among children and adolescents. In other words, schools serve as the primary platform for implementing PA and health education programs, and school-based physical education is a crucial pathway to fostering students’ physical and mental well-being. It not only enhances health awareness but also cultivates healthy lifestyle habits.

International experiences support this perspective. For example, UNESCO’s Quality Physical Education: Guidelines for Policy-Makers (22), the United States’ Comprehensive School Physical Activity Programs (CSPAP) (23), Australia’s Sporting School Program (24), and Canada’s Quality Daily Physical Education (QDPE) (25) all emphasize school-based physical education as a central component in promoting PA among children and adolescents.

This dimension encompasses seven primary indicators: classroom instruction, extracurricular activities, teacher workforce, facilities and equipment, school sports culture, organizational management, and institutional guarantees. Therefore, in implementing PA promotion strategies for children and adolescents, efforts should focus on optimizing and strengthening these seven areas. By adopting differentiated intervention strategies, schools can effectively enhance PA levels across different times of day, educational stages, and gender groups, thereby laying a stronger foundation for the comprehensive development and health of children and adolescents.

### 4.2 Family involvement

Families play a crucial role in promoting PA among children and adolescents, as a supportive home environment for exercise can effectively foster their overall development (26). A study on the characteristics of sports environments for Chinese children and adolescents revealed that families exert the strongest influence on participation in PA, and that the combined effect of family and school is greater than that of family and community (27).

In this study, the family dimension encompasses three primary indicators: parental attitude, parental behavior, and family atmosphere. Parents are key drivers of behavior change in children and adolescents; inactive parents are likely to raise inactive children, while active parental behaviors can help children establish regular PA habits (28). Such influences include parental socialization, role modeling, and active support. Research has shown that if both parents are physically active, their children are 5.8 times more likely to be active than their peers. Conversely, children of inactive parents are six times more likely to be inactive (29, 30).

The theoretical model of Parental Engagement, Support, Physical Activity, and Academic Performance (PESPAAP) proposed by Ryan D. Burns and colleagues also highlights that parental participation positively influences both PA levels and academic performance in children and adolescents (31). However, surveys on the development of family support environments for PA in China indicate that parents often provide insufficient behavioral support for their children’s PA participation, with “more preaching than action and a lack of companionship” being common issues that urgently need improvement (32).

Therefore, in designing strategies to promote PA among children and adolescents, it is crucial to highlight the role of parents as behavioral role models and to cultivate a supportive family environment for PA. Encouraging parents to actively participate in PA alongside their children represents a key pathway for improving PA levels in this population.

### 4.3 Community support

Community-based interventions have proven effective in increasing PA across populations (33). For children and adolescents, in particular, a considerable share of their PA takes place in the immediate vicinity of their homes (34). As critical settings for social interaction, social integration, and healthy development through sport, communities are increasingly recognized as essential platforms for delivering public sport services and as fundamental units of youth sport governance (35). Implementing community-based sports programs is widely regarded as a primary strategy for promoting PA among children and adolescents, supported by initiatives such as facilitating youth participation in organized activities, enhancing community sport facilities, and expanding accessible spaces for PA.

In this study, the community dimension includes four primary indicators: sports facilities, built environment, health services, and organizational management. Research has shown that community designs and features that support PA—such as safe and accessible walking and cycling infrastructure, as well as other favorable built environment elements—are more effective in promoting participation in leisure-time PA for children and adolescents compared to environments lacking these features (36). Accordingly, factors such as pedestrian infrastructure (e.g., sidewalk availability), street design (e.g., connectivity), GIS-measured environmental features, self-reported neighborhood characteristics, walkability indices, availability of park exercise equipment, and accessibility of recreational fitness centers have all emerged as important determinants of PA. These factors are also key intervention measures for increasing children’s and adolescents’ engagement in PA at the community level (37).

### 4.4 Societal collaboration

As efforts to promote PA among children and adolescents advance, leveraging social support, introducing corporate sponsorship, and integrating sports services from non-profit organizations through school–enterprise collaborations have gradually become important approaches. Research indicates that policy-driven, multicomponent, school-based PA interventions can enhance activity levels and help prevent obesity among Chinese children and adolescents (38). The Expert Consensus on Physical Activity and Health in Chinese Children and Adolescents (2020) highlighted that, despite extensive evidence of the benefits of PA, PA promotion has not received sufficient attention as a public health priority from government, schools, or communities in China (12).

The promotion of PA in children and adolescents depends not only on policy support and institutional guarantees from the government, but also on strong support and collaboration from all sectors of society. Efforts should include fostering a public culture that values sports, shaping positive social discourse, and creating enabling material environments to collectively improve PA levels. In this study, the society dimension comprises three primary indicators: institutional guarantees, environmental development, and resource integration.

As the outermost layer of the socio-ecological model, governmental resources, power distribution, and regulatory systems fundamentally shape the social determinants of PA (39). In recent years, the Chinese government has introduced a series of youth sports and health promotion policies, providing institutional guarantees for the high-quality development of youth sports. Environmental development for PA promotion emphasizes integrating PA into daily life, embedding it into multiple aspects of children’s and adolescents’ routines, and fostering a cultural and ecological environment where society, organizations, and all population groups participate collectively.

Moreover, efforts should encompass the broader educational ecosystem in which children and adolescents are situated, integrating policy and institutional resources, event resources, financial support, information, technology, platforms, and cultural resources from schools, families, communities, government agencies, sports enterprises, and social organizations. By transcending temporal and spatial barriers and breaking regional limitations, a collaborative model can be established—featuring government-led macro-level regulation alongside coordinated participation from schools, families, communities, sports organizations, and professional associations—thus achieving a whole-of-society approach to promoting PA among children and adolescents.

### 4.5 Strengths and limitations

This study developed a holistic framework for promoting PA among children and adolescents, offering both theoretical and practical value. Its key strength lies in providing clear stakeholder responsibilities across schools, families, communities, and society, combined with weighted indicators that shift PA promotion from experience-based to evidence-based decision-making. The integration of socio-ecological, behavior change, and social cognitive theories further enhances theoretical robustness. Importantly, the study demonstrates methodological complementarity: while statistical thresholds ensured rigor, some indicators with weaker numerical results were retained due to consistent expert reasoning, strengthening contextual fit. Although developed in China’s sociocultural and policy context, the methodological approach may inform similar efforts internationally.

Compared with international tools such as the Canadian Assessment of Physical Literacy (CAPL) and the Comprehensive School Physical Activity Program (CSPAP) (40, 41), this framework adopts a broader socio-ecological perspective by incorporating four domains—schools, families, communities, and society—and establishes operationalized, weighted indicators absent in prior Chinese guidelines (11, 12).

Nevertheless, several limitations should be acknowledged. The weighting process in AHP inevitably involves a degree of subjectivity, as it relies on expert judgments even when consistency checks are applied. The composition of the expert panel—primarily Chinese scholars from closely related disciplines—may also constrain disciplinary breadth and international representativeness, potentially introducing bias. In addition, although the two-round Delphi process balanced rigor with feasibility, a small number of indicators were retained despite relatively low FF values or higher CVs in Round 2; while theoretically important, this decision may reduce reproducibility. Moreover, the framework has not yet been empirically validated, limiting its immediate generalizability. Macro-level influences such as economic conditions and cultural norms were likewise not explicitly incorporated, despite their potential importance for shaping PA behaviors. Finally, as the framework is based largely on expert consensus, future research should combine large-scale social surveys and empirical validation to enhance its reliability, validity, and practical applicability.

## 5 Conclusion

This study, employing a modified Delphi method and AHP, represents the first attempt to develop a localized framework for promoting PA among Chinese children and adolescents. The framework, comprising four core dimensions with weighted indicators, situates PA promotion within China’s sociocultural context and addresses a critical research gap. It offers a solid foundation for both evaluating barriers and outcomes and guiding evidence-based intervention strategies. Future research should prioritize developing validated measurement tools grounded in this framework and examining its associations with PA, developmental, and health outcomes. Ultimately, this framework holds promise not only for advancing theoretical understanding and practical application in China but also for informing PA promotion efforts in other contexts.

## Data Availability

The raw data supporting the conclusions of this article will be made available by the authors, without undue reservation.
