# Supplementary material for: Developing a socio-ecological framework for promoting physical activity among Chinese children and adolescents: a Delphi–AHP study
Source: Front Public Health. 2025 Dec 2;13:1700544. doi: 10.3389/fpubh.2025.1700544 (PMC12705577; doi:10.3389/fpubh.2025.1700544)
Supplement: Supplementary file 1 [file Table_1.docx]

**Appendix Table 1. List of Consulation Experts**

| **No.** | **Name** | **Gender** | **Education** | **Title^b^** | **YOPE^a^** | **Research Field** | **Institution (Region)** |
| --- | --- | --- | --- | --- | --- | --- | --- |
| 1 | Guo ** | Male | PhD | Professor | 30 | Physical Education | Department of Education of Henan Province (Henan, Central China) |
| 2 | Guo ** | Male | PhD | Professor | 25 | Adolescent Health Promotion | Capital University of Physical Education and Sports (Beijing, North China) |
| 3 | Ma ** | Female | PhD | Professor | 32 | Adolescent Health Promotion | Institute of Child and Adolescent Health, Peking University (Beijing, North China) |
| 4 | Pan ** | Male | Master | Professor | 35 | Physical Activity and Health | Yangzhou University (Jiangsu, East China) |
| 5 | Shi * | Male | PhD | Professor | 32 | Physical Education | Shaanxi Normal University (Shaanxi, Northwest China) |
| 6 | Sun * | Male | PhD | Professor | 25 | Physical Activity and Health | East China Normal University (Shanghai, East China) |
| 7 | Wang ** | Male | PhD | Professor | 18 | Physical Activity and Health | The Chinese University of Hong Kong (Hong Kong, South China) |
| 8 | Wen * | Male | PhD | Professor | 12 | Physical Activity and Health | Zhejiang University (Zhejiang, East China) |
| 9 | Chen ** | Female | PhD | Associate Professor | 25 | Adolescent Health Promotion | University of Michigan, USA (United States) |
| 10 | Guo * | Male | PhD | Associate Professor | 5 | Physical Activity and Health | Ningbo University (Zhejiang, East China) |
| 11 | Wang * | Male | PhD | Associate Professor | 10 | Physical Activity and Health | Shandong Normal University (Shandong, East China) |
| 12 | Yin ** | Male | PhD | Associate Professor | 8 | Physical Education | East China Normal University (Shanghai, East China) |
| 13 | Gan * | Male | PhD | Associate Professor | 28 | Physical Education | Hubei Academy of Educational Sciences (Hubei, Central China) |
| 14 | Li * | Female | Master | Associate Professor | 12 | Physical Education | Xingchen Junior Middle School, Liangjiang New Area, Chongqing (Chongqing, Southwest China) |
| 15 | Wang ** | Male | Master | Associate Professor | 26 | Physical Education | Shanghai Municipal Education Commission, Teaching and Research Office (Shanghai, East China) |
| 16 | Yang * | Male | Master | Associate Professor | 18 | Physical Education | Nantong Institute of Educational Sciences, Jiangsu Province (Jiangsu, East China) |
| 17 | Jiang * | Female | Bachelor | Associate Professor | 19 | Physical Education | Donghe Branch, Yantian Foreign Language Primary School, Shenzhen (Guangdong, South China) |

a. YOPE: Years of professional experience were calculated up to December 31, 2024.

b. All professional job titles were standardized based on the commonly used academic rank system in China: Teaching Assistant, Assistant Professor, Associate Professor, and Professor.

**Appendix Table 2. Summary of Expert Consultation Results in the First Round**

| **Indicator Name** | **M** | **SD** | **CV** | **FF** | **M ≥ M-2×SD** | **CV ≤ CV+2×SD** | **FF ≥ FF-2×SD** |
| --- | --- | --- | --- | --- | --- | --- | --- |
| 1 School Leadership | 4.9412 | 0.2500 | 0.9375 | 0.0506 | 4.4412 | 0.5506 | 0.4375 |
| 2 Family Involvement | 5.0000 | 0.0000 | 1.0000 | 0.0000 | 5.0000 | 0.0000 | 1.0000 |
| 3 Community Support | 4.3529 | 0.6191 | 0.4375 | 0.1422 | 3.1147 | 1.3805 | -0.8008 |
| 4 Government Support | 4.5294 | 0.7303 | 0.6250 | 0.1612 | 3.0688 | 1.6218 | -0.8356 |
| 5 Societal Collaboration | 4.2941 | 0.6021 | 0.3750 | 0.1402 | 3.0900 | 1.3444 | -0.8292 |
| 1.1 Classroom Instruction | 5.0000 | 0.0000 | 1.0000 | 0.0000 | 5.0000 | 0.0000 | 1.0000 |
| 1.2 Extracurricular Activities | 4.8824 | 0.2500 | 0.9375 | 0.0512 | 4.3824 | 0.5512 | 0.4375 |
| 1.3 Teacher Workforce | 4.7647 | 0.4472 | 0.7500 | 0.0939 | 3.8703 | 0.9883 | -0.1444 |
| 1.4 Facilities & Equipment | 4.7059 | 0.6021 | 0.7500 | 0.1279 | 3.5017 | 1.3321 | -0.4542 |
| 1.5 School Sports Culture | 4.5882 | 0.8139 | 0.6875 | 0.1774 | 2.9604 | 1.8053 | -0.9404 |
| 1.6 Organizational Management | 4.7059 | 0.4787 | 0.6875 | 0.1017 | 3.7485 | 1.0592 | -0.2699 |
| 2.1 Parental Attitudes | 4.8824 | 0.2500 | 0.9375 | 0.0512 | 4.3824 | 0.5512 | 0.4375 |
| 2.2 Parental Behaviors | 5.0000 | 0.0000 | 1.0000 | 0.0000 | 5.0000 | 0.0000 | 1.0000 |
| 2.3 Family Atmosphere | 4.7059 | 0.4787 | 0.6875 | 0.1017 | 3.7485 | 1.0592 | -0.2699 |
| 3.1 Sports Facilities | 4.7059 | 0.4787 | 0.6875 | 0.1017 | 3.7485 | 1.0592 | -0.2699 |
| 3.2 Sports Environment | 4.1765 | 0.6551 | 0.3125 | 0.1569 | 2.8663 | 1.4671 | -0.9977 |
| 3.3 Health Services | 4.2353 | 0.8563 | 0.4375 | 0.2022 | 2.5226 | 1.9149 | -1.2752 |
| 3.4 Organizational Management | 4.2941 | 0.6021 | 0.3750 | 0.1402 | 3.0900 | 1.3444 | -0.8292 |
| 4.1 Institutional Guarantee | 4.7647 | 0.5774 | 0.8125 | 0.1212 | 3.6100 | 1.2759 | -0.3422 |
| 4.2 Environmental Development | 4.5882 | 0.6292 | 0.6250 | 0.1371 | 3.3299 | 1.3954 | -0.6333 |
| 5.1 Incentive Mobilization | 4.4118 | 0.6292 | 0.5000 | 0.1426 | 3.1535 | 1.4009 | -0.7583 |
| 5.2 Resource Integration | 4.4706 | 0.7303 | 0.6250 | 0.1634 | 3.0100 | 1.6239 | -0.8356 |
| 1.1.1 PE and Health Classes | 5.0000 | 0.0000 | 1.0000 | 0.0000 | 5.0000 | 0.0000 | 1.0000 |
| 1.1.2 Micro-activities in Academic Classes | 3.8824 | 0.6191 | 0.1250 | 0.1595 | 2.6441 | 1.3978 | -1.1133 |
| 1.1.3 Cultural and Sports Integrated Curriculum | 4.1176 | 0.7719 | 0.3125 | 0.1875 | 2.5738 | 1.7313 | -1.2313 |
| 1.2.1 Morning Exercises | 4.4118 | 0.6191 | 0.4375 | 0.1403 | 3.1735 | 1.3786 | -0.8008 |
| 1.2.2 Breaktime Exercises | 4.6471 | 0.6191 | 0.6875 | 0.1332 | 3.4088 | 1.3715 | -0.5508 |
| 1.2.3 Sports Training | 4.4118 | 0.7188 | 0.5000 | 0.1629 | 2.9742 | 1.6005 | -0.9376 |
| 1.2.4 Sports Competitions | 4.6471 | 0.4787 | 0.6875 | 0.1030 | 3.6896 | 1.0604 | -0.2699 |
| 1.2.5 Sports Interest Groups/Clubs/Associations | 4.8824 | 0.3416 | 0.8750 | 0.0700 | 4.1992 | 0.7531 | 0.1919 |
| 1.2.6 Sports Festivals/Sports Meets | 4.5294 | 0.6325 | 0.5625 | 0.1396 | 3.2645 | 1.4045 | -0.7024 |
| 1.2.7 Parent–School Collaborative Sports Activities | 4.4118 | 0.6191 | 0.4375 | 0.1403 | 3.1735 | 1.3786 | -0.8008 |
| 1.3.1 Teacher Quantity | 4.4118 | 0.5000 | 0.3750 | 0.1133 | 3.4118 | 1.1133 | -0.6250 |
| 1.3.2 Teacher Quality | 4.8235 | 0.4031 | 0.8125 | 0.0836 | 4.0173 | 0.8898 | 0.0063 |
| 1.4.1 Facility Provision | 4.8235 | 0.4031 | 0.8125 | 0.0836 | 4.0173 | 0.8898 | 0.0063 |
| 1.4.2 Facility Safety | 4.7059 | 0.6021 | 0.7500 | 0.1279 | 3.5017 | 1.3321 | -0.4542 |
| 1.4.3 Facility Accessibility | 4.5294 | 0.5123 | 0.5625 | 0.1131 | 3.5047 | 1.1378 | -0.4622 |
| 1.4.4 Facility Maintenance | 4.3529 | 0.6191 | 0.4375 | 0.1422 | 3.1147 | 1.3805 | -0.8008 |
| 1.5.1 Sports Activity Atmosphere | 4.7647 | 0.4472 | 0.7500 | 0.0939 | 3.8703 | 0.9883 | -0.1444 |
| 1.5.2 Sports Information and Publicity | 4.2941 | 0.5774 | 0.3125 | 0.1345 | 3.1394 | 1.2892 | -0.8422 |
| 1.5.3 Teacher–Student Sports Awareness | 4.7059 | 0.7042 | 0.8125 | 0.1496 | 3.2976 | 1.5579 | -0.5958 |
| 1.6.1 Organizational Structure | 4.6471 | 0.6191 | 0.6875 | 0.1332 | 3.4088 | 1.3715 | -0.5508 |
| 1.6.2 Work Systems and Plans | 4.5882 | 0.6292 | 0.6250 | 0.1371 | 3.3299 | 1.3954 | -0.6333 |
| 1.6.3 Financial Support | 4.7059 | 0.4787 | 0.6875 | 0.1017 | 3.7485 | 1.0592 | -0.2699 |
| 1.6.4 Monitoring and Feedback | 4.7059 | 0.4787 | 0.6875 | 0.1017 | 3.7485 | 1.0592 | -0.2699 |
| 1.6.5 Evaluation and Incentives | 4.4706 | 0.6292 | 0.5000 | 0.1407 | 3.2123 | 1.3990 | -0.7583 |
| 2.1.1 Verbal Encouragement | 4.7647 | 0.4472 | 0.7500 | 0.0939 | 3.8703 | 0.9883 | -0.1444 |
| 2.1.2 Active Concern | 4.5294 | 0.6325 | 0.5625 | 0.1396 | 3.2645 | 1.4045 | -0.7024 |
| 2.1.3 Communication of Benefits | 4.3529 | 0.6021 | 0.3750 | 0.1383 | 3.1488 | 1.3425 | -0.8292 |
| 2.2.1 Parents’ Own Participation | 4.5294 | 0.6325 | 0.5625 | 0.1396 | 3.2645 | 1.4045 | -0.7024 |
| 2.2.2 Joint Parent–Child Participation | 4.9412 | 0.2500 | 0.9375 | 0.0506 | 4.4412 | 0.5506 | 0.4375 |
| 2.2.3 Parents Observing Children’s Participation | 4.1765 | 0.8062 | 0.3125 | 0.1930 | 2.5640 | 1.8055 | -1.3000 |
| 2.3.1 Financial Support for Sports | 4.5294 | 0.5123 | 0.5625 | 0.1131 | 3.5047 | 1.1378 | -0.4622 |
| 2.3.2 Space Provision for Activities | 4.2353 | 0.6831 | 0.3750 | 0.1613 | 2.8690 | 1.5276 | -0.9913 |
| 2.3.3 Limiting Sedentary Behaviors | 4.2353 | 0.6551 | 0.3125 | 0.1547 | 2.9251 | 1.4649 | -0.9977 |
| 3.1.1 Public Sports Facilities | 4.4706 | 0.5164 | 0.5000 | 0.1155 | 3.4378 | 1.1483 | -0.5328 |
| 3.1.2 Commercial Fitness Clubs | 3.8235 | 0.6551 | 0.1250 | 0.1713 | 2.5133 | 1.4816 | -1.1852 |
| 3.1.3 Community Children’s Activity Centers | 4.7059 | 0.4787 | 0.6875 | 0.1017 | 3.7485 | 1.0592 | -0.2699 |
| 3.2.1 Community Open Space Utilization | 3.9412 | 0.7719 | 0.2500 | 0.1959 | 2.3974 | 1.7397 | -1.2938 |
| 3.2.2 Community Safety | 4.2941 | 0.7746 | 0.4375 | 0.1804 | 2.7449 | 1.7296 | -1.1117 |
| 3.2.3 Daily PA Signage | 3.8235 | 0.6831 | 0.1250 | 0.1787 | 2.4573 | 1.5449 | -1.2413 |
| 3.2.4 Walkability of Community Roads | 3.8824 | 0.6551 | 0.1250 | 0.1687 | 2.5721 | 1.4790 | -1.1852 |
| 3.2.5 Accessibility of Fitness Venues | 4.5882 | 0.5123 | 0.5625 | 0.1117 | 3.5635 | 1.1364 | -0.4622 |
| 3.3.1 Health Education and Counseling | 4.1765 | 0.8062 | 0.3750 | 0.1930 | 2.5640 | 1.8055 | -1.2375 |
| 3.3.2 Health Skills Guidance/Training | 4.3529 | 0.7042 | 0.4375 | 0.1618 | 2.9446 | 1.5701 | -0.9708 |
| 3.3.3 Organization of Sports Activities | 4.4706 | 0.7274 | 0.5625 | 0.1627 | 3.0157 | 1.6176 | -0.8924 |
| 3.3.4 Health Information Dissemination | 4.1176 | 0.8539 | 0.3750 | 0.2074 | 2.4098 | 1.9152 | -1.3328 |
| 3.4.1 Community Sports Organizations | 4.1176 | 0.6191 | 0.2500 | 0.1504 | 2.8794 | 1.3886 | -0.9883 |
| 3.4.2 Community Sports Regulations | 3.8000 | 0.8062 | 0.2500 | 0.2122 | 2.1875 | 1.8246 | -1.3625 |
| 4.1.1 Policy Support | 4.6667 | 0.4787 | 0.6875 | 0.1026 | 3.7092 | 1.0600 | -0.2699 |
| 4.1.2 Legal Constraints | 4.6000 | 0.5000 | 0.6250 | 0.1087 | 3.6000 | 1.1087 | -0.3750 |
| 4.1.3 Financial Support | 4.6667 | 0.4787 | 0.6875 | 0.1026 | 3.7092 | 1.0600 | -0.2699 |
| 4.1.4 Supervision and Evaluation | 4.4667 | 0.6325 | 0.5625 | 0.1416 | 3.2018 | 1.4065 | -0.7024 |
| 4.1.5 Information Disclosure | 4.0667 | 0.6191 | 0.2500 | 0.1522 | 2.8284 | 1.3905 | -0.9883 |
| 4.2.1 Cultural Environment | 4.3333 | 0.6191 | 0.4375 | 0.1429 | 3.0951 | 1.3812 | -0.8008 |
| 4.2.2 Ecological Environment | 4.3333 | 0.6191 | 0.4375 | 0.1429 | 3.0951 | 1.3812 | -0.8008 |
| 5.1.1 Public Awareness | 4.4000 | 0.5123 | 0.4375 | 0.1164 | 3.3753 | 1.1411 | -0.5872 |
| 5.1.2 Group Action Support | 4.2667 | 0.4787 | 0.3125 | 0.1122 | 3.3092 | 1.0696 | -0.6449 |
| 5.2.1 Facility & Equipment Resources | 4.5333 | 0.5123 | 0.5625 | 0.1130 | 3.5086 | 1.1377 | -0.4622 |
| 5.2.2 Human Resources | 4.4667 | 0.6325 | 0.5625 | 0.1416 | 3.2018 | 1.4065 | -0.7024 |
| 5.2.3 Financial Resources | 4.4667 | 0.6325 | 0.5625 | 0.1416 | 3.2018 | 1.4065 | -0.7024 |
| 5.2.4 Information Resources | 4.0000 | 0.5737 | 0.1875 | 0.1434 | 2.8525 | 1.2909 | -0.9600 |
| 5.2.5 Technological Resources | 4.2000 | 0.6831 | 0.3750 | 0.1627 | 2.8337 | 1.5289 | -0.9913 |

**Appendix Table 3. Indicator Weights of the Framework for Promoting Physical Activity among Chinese Children and Adolescents**

| **Dimension Indicator** | **Weight (Wi)** | **Primary Indicator (17 items)** | **Weight (Wi)** | **Secondary Indicator (58 items)** | **Weight (Wi)** |
| --- | --- | --- | --- | --- | --- |
| 1 School Leadership | 0.5437 | 1.1 Classroom Instruction | 0.2254 | 1.1.1 PE and Health Classes | 0.1447 |
|  |  |  |  | 1.1.2 Micro-activities in Academic Classes | 0.0383 |
|  |  |  |  | 1.1.3 Interdisciplinary Integrated PE Curriculum | 0.0424 |
|  |  | 1.2 Extracurricular Activities | 0.1546 | 1.2.1 Morning Physical Activities | 0.0182 |
|  |  |  |  | 1.2.2 Extended Recess Physical Activities | 0.0378 |
|  |  |  |  | 1.2.3 After-school Training/Competitions | 0.0283 |
|  |  |  |  | 1.2.4 Sports Interest Groups/Clubs/Associations | 0.0265 |
|  |  |  |  | 1.2.5 Sports Festivals/Sports Meets | 0.0263 |
|  |  |  |  | 1.2.6 Parent–School Collaborative Activities | 0.0176 |
|  |  | 1.3 Teacher Workforce | 0.2208 | 1.3.1 Teacher Quantity | 0.1057 |
|  |  |  |  | 1.3.2 Teacher Quality | 0.1152 |
|  |  | 1.4 Facilities & Equipment | 0.1202 | 1.4.1 Facility Provision | 0.0521 |
|  |  |  |  | 1.4.2 Facility Safety | 0.0457 |
|  |  |  |  | 1.4.3 Facility Accessibility | 0.0681 |
|  |  | 1.5 School Sports Culture | 0.0804 | 1.5.1 Sports Activity Atmosphere | 0.036 |
|  |  |  |  | 1.5.2 Sports Information and Publicity | 0.0128 |
|  |  |  |  | 1.5.3 Teacher–Student Sports Awareness | 0.0316 |
|  |  | 1.6 Organizational Management | 0.0958 | 1.6.1 Working Group | 0.0234 |
|  |  |  |  | 1.6.2 Work Systems and Plans | 0.0287 |
|  |  |  |  | 1.6.3 Evaluation and Incentives | 0.0437 |
|  |  | 1.7 Institutional Guarantee | 0.1027 | 1.7.1 Financial Support | 0.077 |
|  |  |  |  | 1.7.2 Monitoring and Feedback | 0.0258 |
| 2 Family Involvement | 0.2314 | 2.1 Parental Attitude | 0.2645 | 2.1.1 Verbal Encouragement | 0.0457 |
|  |  |  |  | 2.1.2 Active Concern | 0.052 |
|  |  |  |  | 2.1.3 Communication of Benefits | 0.0699 |
|  |  |  |  | 2.1.4 Time Support | 0.0968 |
|  |  | 2.2 Parental Behavior | 0.4235 | 2.2.1 Parents’ Own Participation | 0.0513 |
|  |  |  |  | 2.2.2 Joint Parent–Child Participation | 0.2819 |
|  |  |  |  | 2.2.3 Parents Guiding Children’s Participation | 0.0904 |
|  |  | 2.3 Family Atmosphere | 0.312 | 2.3.1 Financial Support for Sports | 0.1398 |
|  |  |  |  | 2.3.2 Space Provision for Activities | 0.0676 |
|  |  |  |  | 2.3.3 Limiting Sedentary Behaviors | 0.1046 |
| 3 Community Support | 0.1211 | 3.1 Sports Facilities | 0.2597 | 3.1.1 Public Sports Facilities | 0.1105 |
|  |  |  |  | 3.1.2 Youth Sports Clubs | 0.0361 |
|  |  |  |  | 3.1.3 Community Children’s Activity Centers | 0.1132 |
|  |  | 3.2 Built Environment | 0.3169 | 3.2.1 Community Space Utilization | 0.0373 |
|  |  |  |  | 3.2.2 Community Safety | 0.0967 |
|  |  |  |  | 3.2.3 Motivational Signage for PA | 0.0321 |
|  |  |  |  | 3.2.4 Community Walkability | 0.0419 |
|  |  |  |  | 3.2.5 Accessibility of Community Sports Venues | 0.109 |
|  |  | 3.3 Health Services | 0.2412 | 3.3.1 Health Education and Counseling | 0.0396 |
|  |  |  |  | 3.3.2 Health Skills Guidance/Training | 0.0718 |
|  |  |  |  | 3.3.3 Organization of Sports Activities | 0.0982 |
|  |  |  |  | 3.3.4 Health Information Dissemination | 0.0316 |
|  |  | 3.4 Organizational Management | 0.1821 | 3.4.1 Community Sports Institutions | 0.1269 |
|  |  |  |  | 3.4.2 Community Sports Support System | 0.0552 |
| 4 Societal Collaboration | 0.1038 | 4.1 Institutional Guarantee | 0.4899 | 4.1.1 Policy Support | 0.0971 |
|  |  |  |  | 4.1.2 Legal Constraints | 0.0693 |
|  |  |  |  | 4.1.3 Financial Support | 0.1653 |
|  |  |  |  | 4.1.4 Supervision and Evaluation | 0.1175 |
|  |  |  |  | 4.1.5 Information Disclosure | 0.0406 |
|  |  | 4.2 Environmental Development | 0.1688 | 4.2.1 Cultural Environment | 0.0889 |
|  |  |  |  | 4.2.2 Ecological Environment | 0.08 |
|  |  | 4.3 Resource Integration | 0.3413 | 4.3.1 Facility & Equipment Resources | 0.0819 |
|  |  |  |  | 4.3.2 Human Resources | 0.0745 |
|  |  |  |  | 4.3.3 Financial Resources | 0.1055 |
|  |  |  |  | 4.3.4 Information Resources | 0.0313 |
|  |  |  |  | 4.3.5 Technological Resources | 0.0481 |

**Appendix Table 4. Summary of Consistency Ratios (CR) for All Matrices**

|  | **Number of Matrices** | **Acceptable CR (≤0.10)** | **CR > 0.10 (Corrected)** | **CR > 0.10 (Excluded)** | **Final Included** |
| --- | --- | --- | --- | --- | --- |
| **Dimension-level (4 domains)** | 17 | 17 | 0 | 0 | 17 |
| **School Leadership** | 17 | 17 | 0 | 0 | 17 |
| **Family Involvement** | 17 | 17 | 0 | 0 | 17 |
| **Community Support** | 17 | 17 | 0 | 0 | 17 |
| **Societal Collaboration** | 17 | 17 | 0 | 0 | 17 |
| **Total** | 85 | 85 | 0 | 0 | 85 |

**Appendix Table 5. Pairwise Comparison Matrix for Dimensions of the Preschool PL Framework**

|  | **School Leadership** | **Family Involvement** | **Community Support** | **Societal Collaboration** | **Mean** | **CR** |
| --- | --- | --- | --- | --- | --- | --- |
| **School Leadership** | 1.00 | 2.24 | 4.49 | 3.97 | 3.00 | 0.08 |
| **Family Involvement** | 0.45 | 1.00 | 2.01 | 1.77 | 2.85 |  |
| **Community Support** | 0.22 | 0.50 | 1.00 | 0.88 | 1.19 |  |
| **Societal Collaboration** | 0.25 | 0.56 | 1.13 | 1.00 | 2.96 |  |

**Appendix Table 6. Pairwise Comparison Matrix for the First-Level Indicators of “School Leadership” Dimension**

|  | **Classroom Instruction** | **Extracurricular Activities** | **Teacher Workforce** | **Facilities & Equipment** | **School Sports Culture** | **Organizational Management** | **Institutional Guarantee** | **Mean** | **CR** |
| --- | --- | --- | --- | --- | --- | --- | --- | --- | --- |
| **Classroom Instruction** | 1.00 | 1.48 | 1.13 | 2.12 | 2.32 | 2.40 | 2.15 | 5.00 | 0.09 |
| **Extracurricular Activities** | 0.68 | 1.00 | 0.76 | 1.43 | 1.57 | 1.62 | 1.46 | 4.94 |  |
| **Teacher Workforce** | 0.88 | 1.31 | 1.00 | 1.88 | 2.05 | 2.12 | 1.90 | 4.88 |  |
| **Facilities & Equipment** | 0.47 | 0.70 | 0.53 | 1.00 | 1.09 | 1.13 | 1.02 | 4.59 |  |
| **School Sports Culture** | 0.43 | 0.64 | 0.49 | 0.92 | 1.00 | 1.03 | 0.93 | 4.41 |  |
| **Organizational Management** | 0.42 | 0.62 | 0.47 | 0.89 | 0.97 | 1.00 | 0.90 | 4.53 |  |
| **Institutional Guarantee** | 0.46 | 0.69 | 0.53 | 0.99 | 1.08 | 1.11 | 1.00 | 4.13 |  |

**Appendix Table 7. Pairwise Comparison Matrix for the First-Level Indicators of “Family Involvement” Dimension**

|  | **Parental Attitudes** | **Parental Behaviors** | **Family Atmosphere** | **Mean** | **CR** |
| --- | --- | --- | --- | --- | --- |
| **Parental Attitudes** | 1.00 | 0.55 | 0.76 | 4.94 | 0.06 |
| **Parental Behaviors** | 1.80 | 1.00 | 1.37 | 4.94 |  |
| **Family Atmosphere** | 1.31 | 0.73 | 1.00 | 4.35 |  |

**Appendix Table 8. Pairwise Comparison Matrix for the First-Level Indicators of “Community Support” Dimension**

|  | **Sports Facilities** | **Built Environment** | **Health Services** | **Organizational Management** | **Mean** | **CR** |
| --- | --- | --- | --- | --- | --- | --- |
| **Sports Facilities** | 1.00 | 0.93 | 1.33 | 1.45 | 4.59 | 0.09 |
| **Built Environment** | 1.08 | 1.00 | 1.43 | 1.57 | 3.88 |  |
| **Health Services** | 0.75 | 0.70 | 1.00 | 1.09 | 3.82 |  |
| **Organizational Management** | 0.69 | 0.64 | 0.91 | 1.00 | 3.88 |  |

**Appendix Table 9. Pairwise Comparison Matrix for the First-Level Indicators of “Societal Collaboration” Dimension**

|  | **Institutional Guarantee** | **Environmental Development** | **Resource Integration** | **Mean** | **CR** |
| --- | --- | --- | --- | --- | --- |
| **Institutional Guarantee** | 1.00 | 2.90 | 1.47 | 4.53 | 0.02 |
| **Environmental Development** | 0.34 | 1.00 | 0.51 | 4.41 |  |
| **Resource Integration** | 0.68 | 1.98 | 1.00 | 3.59 |  |

**Appendix Table 10. Example of Individual Pairwise Comparison Matrix (Expert #12, anonymized)**

|  | **Sports Facilities** | **Built Environment** | **Health Services** | **Organizational Management** | **CR** |
| --- | --- | --- | --- | --- | --- |
| **Sports Facilities** | 1.00 | 0.17 | 0.20 | 0.25 | 0.09 |
| **Built Environment** | 6.00 | 1.00 | 4.00 | 6.00 |  |
| **Health Services** | 5.00 | 0.25 | 1.00 | 2.00 |  |
| **Organizational Management** | 4.00 | 0.17 | 0.50 | 1.00 |  |

Note: This example is an anonymized individual matrix provided for illustrative purposes only. The expert’s CR = 0.09 (≤0.10), and therefore it was included in the final analysis.
